# Hematological and hemorheological Determinants of the Six-Minute Walk Test Performance in Children with Sickle Cell Anemia

**DOI:** 10.1371/journal.pone.0077830

**Published:** 2013-10-17

**Authors:** Xavier Waltz, Marc Romana, Marie-Dominique Hardy-Dessources, Yann Lamarre, Lydia Divialle-Doumdo, Marie Petras, Vanessa Tarer, Régine Hierso, Kizzy-Clara Baltyde, Benoît Tressières, Marie-Laure Lalanne-Mistrih, Fréderic Maillard, Olivier Hue, Maryse Etienne-Julan, Philippe Connes

**Affiliations:** 1 UMR Inserm 665, Université des Antilles et de la Guyane, Pointe-à-Pitre, Guadeloupe, France; 2 Laboratoire ACTES (EA 3596), Département de Physiologie, Université des Antilles et de la Guyane, Pointe-à-Pitre, Guadeloupe, France; 3 Laboratory of Excellence GR-Ex “The red cell: from genesis to death”, PRES Sorbonne Paris Cité, Paris, France; 4 Unité Transversale de la Drépanocytose, Centre Hospitalier et Universitaire de Pointe-à-Pitre, Pointe-à-Pitre, Guadeloupe, France; 5 Centre de référence maladies rares pour la drépanocytose aux Antilles-Guyane, Centre Hospitalier et Universitaire de Pointe-à-Pitre, Pointe-à-Pitre, Guadeloupe, France; 6 CIC-EC 802 Inserm, Centre Hospitalier Universitaire de Pointe-à-Pitre, Pointe-à-Pitre, Guadeloupe, France; 7 Service de Pédiatrie du Centre Hospitalier et Universitaire de Pointe-à-Pitre, Pointe-à-Pitre, Guadeloupe, France; 8 Institut Universitaire de France, Paris, France; Emory University/Georgia Insititute of Technology, United States of America

## Abstract

The six-minute walk test is a well-established submaximal exercise reflecting the functional status and the clinical severity of sickle cell patients. The aim of the present cross-sectional study was to investigate the biological determinants of the six-minute walk test performance in children with sickle cell anemia. Hematological and hemorheological parameters, pulmonary function and the six-minute walk test performance were determined in 42 children with sickle cell anemia at steady state. The performance during the six-minute walk test was normalized for age, sex and height and expressed as percentage of the predicted six-minute walk distance. We showed that a high level of anemia, a low fetal hemoglobin expression and low red blood cell deformability were independent predictors of a low six-minute walk test performance. This study describes for the first time the impact of blood rheology in the six-minute walk test performance in children with sickle cell anemia.

## Introduction

The six-minute walk test (6MWT) is a submaximal exercise test used in several diseases, and more recently in patients with sickle cell anemia (SCA), to determine the functional status, physical fitness and the degree of clinical severity in this disease [1]. Machado et al [2] previously reported that the 6MWT distance directly correlated with peak oxygen consumption in SCA. Moreover, the 6MWT performance inversely correlated with the severity of pulmonary hypertension in SCA [3] and pulmonary hypertension therapy improved walk distance [2]. So far, very few studies have focused on the determinants of the 6MWT performance in SCA. 

SCA is characterized by severe hemorheological abnormalities, which play a role in the pathophysiology of several complications [4–10]. In isolated vessels or animals models, a reduction in red blood cell (RBC) deformability and abnormal RBC aggregation properties have been shown to affect microcirculatory blood flow and tissue perfusion [11–14].. Recently, associations between blood rheological alterations and decreased tissue oxygenation, both at the muscle and cerebral level, have been described in sickle cell patients [15,16]. Based on these previous studies, one could suggest that the degree of hemorheological abnormalities, in association with the degree of anemia, could affect the 6MWT performance in SCA population. 

The aim of the present study was to test the associations between hemorheological/hematological abnormalities and the performance during the 6MWT in children with SCA.

## Methods

### Patients

The study included 42 SCA (HbSS) children (8-17 years old) at steady state: no blood transfusions in the previous three months, absence of acute episodes (infection, vaso-occlusive crises (VOC), acute chest syndrome (ACS), stroke, priapism, splenic sequestration) at least one month before inclusion into the study. None of the SCA children was under hydroxyurea treatment. 

Charts were retrospectively reviewed by three physicians to recognize all ACS and VOC episodes from birth to the time of blood sampling based on previously described criteria [8]. An acute event was considered a VOC if the painful episode lasted for more than four hours, the patient felt that the pain was typical of that of vaso-occlusion, no other etiology of pain could be identified by the physicians, and the patient was admitted to the Accident and Emergency Pediatric Department to treat the pain with parenteral opioids (with or without non-steroidal anti-inflammatory drugs). ACS, splenic or hepatic sequestration or exacerbations of chronic painful conditions such as avascular necrosis or leg ulcer were not considered as painful VOC episodes. ACS was defined as the appearance of a new infiltrate on chest radiograph, associated with one or more clinical symptoms such as chest pain, respiratory distress, fever and cough. The rates of ACS and VOC were calculated for each child by dividing the total number of ACS or painful VOC episodes by the number of patient-years [8,17].

The study was conducted in accordance to the Declaration of Helsinki and was approved by the Regional Ethics Committee (CPP Sud/Ouest Outre Mer III, Bordeaux, France, registration number: 2009-A00211-56). Children and their parents were informed of the purpose and procedures of the study, and gave written informed consent. 

### Hematological and hemorheological measurements

Blood was drawn by venipuncture into EDTA tubes and used for measurements of hematological parameters as previously described [8]. Serum lactate dehydrogenase and total bilirubin concentrations were determined by standard biochemical methods.

Hemorheological parameters were measured immediately after sampling and after full re-oxygenation of the blood [18]. Blood viscosity was measured at native hematocrit (Brookfield DVII+ cone-plate viscometer, CPE40-spindle, ^≈^25°C, 90 s^-1^). RBC deformability was determined at 37°C at two shear stresses (3 and 30 Pa) by ecktacytometry (LORCA, RR Mechatronics, Hoorn, The Netherlands). As recommended by the international guidelines in hemorheology [18], RBC aggregation was determined at 37°C via syllectometry (i.e., laser backscatter versus time), (LORCA, RR Mechtronics, Hoom, The Netherlands) and after adjustment of the hematocrit to 40%. Hematocrit standardization was done by removing the adequate volume of plasma after blood centrifugation [18]. The RBC disaggregation threshold, i.e., the minimal shear rate needed to prevent RBC aggregation or to breakdown existing RBC aggregates, was determined using a re-iteration procedure [19].

### Six-minute walk Test (6MWT)

The 6MWT was conducted according to the guidelines of the American Thoracic Society [20]. The percentage of predicted distance was calculated according to the models of Geiger et al [21] which takes into account the sex, height and age. Hemoglobin oxygen saturation (SpO_2_) was obtained by finger pulse oximetry (Sure Signs VS3 No. 3000, Philips Medical System, Andover, MA, USA) before the 6MWT. 

### Pulmonary function tests

Pulmonary function tests were performed on the same day as the blood sampling and the 6MWT (before), and included forced expiratory volume in 1 second (FEV), forced vital capacity (FVC) and FEV/FVC.

### Statistics

All values were expressed as means ± SD. To identify factors associated with the 6MWT performance, we used parametric or non-parametric correlations (Pearson or Spearman, respectively) between the percentage of predicted distance and the others physiological/biological/clinical parameters. Then, all variables at p < 0.20 were included in a multivariate linear regression models to identify the covariates independently associated with the percentage of predicted distance (i.e., 6MWT performance). Significance level was defined as p < 0.05. Analyses were conducted using SPSS (v. 20, IBM SPSS Statistics, Chicago, IL). 

## Results

 The characteristics of SCA children and the results obtained for the correlations between hematological, hemorheological or clinical parameters and the percentage of predicted distance are shown in Table 1. 

**Table 1:** Sickle cell children characteristics and correlation between hematological, hemorheological, and clinical parameters with the percentage of predicted distance.

	**sickle cell children characteristics**	**correlation with the percentage of predicted distance**	**p value**
**Sex ratio (M/F)**	22/20	-	-
**α-thalassemia (%)**	35.7	-	-
**Age (yrs.)**	11.7 ± 2.4	-	-
**Walked distance (m)**	491 ± 64	-	-
**Percentage of predicted distance (%)**	74.5 ± 10.0	-	-
**BMI (Kg.m^-2^)**	16.8 ± 2.3	r = - 0.03	NS
**Heart rate at rest (beat/min)**	82 ± 11	r = 0.16	NS
**MAP (mmHg) §**	79 ± 7	r = - 0.26	0.1
**SpO_2_ (%)**	97.7 ± 2.5	ρ = 0.07	NS
**Fetal hemoglobin (%) §**	8.2 ± 6.4	r = 0.58	< 0.001
**Leukocytes (10^9^.l^-1^) §**	10.7 ± 2.6	r = - 0.22	0.18
**Red blood cells (10^12^.l^-1^) $**	2.9 ± 0.7	r = 0.27	0.10
**Platelets (10^9^.l^-1^) §**	452 ± 125	r = 0.31	< 0.05
**Hemoglobin (g.dl^-1^) $**	8.0 ± 1.3	r = 0.46	< 0.01
**Hematocrit (%) §**	24.7 ± 4.5	r = 0.35	< 0.05
**Mean cell volume (fl)**	79.9 ± 7.7	r = 0.13	NS
**MCH (pg)**	27.5 ± 3.0	ρ = 0.18	NS
**Reticulocytes (%**)	10.7 ± 5.9	ρ = - 0.08	NS
**Lactate dehydrogenase (IU) §**	578 ± 170	ρ = - 0.31	0.06
**Total bilirubin** (**μmol.l^-1^**)	64.2 ± 43.7	ρ = - 0.15	NS
**Blood viscosity (mPa.s^-1^)**	7.0 ± 2.1	r = 0.13	NS
**RBC deformability at 3 Pa (a.u) §**	0.16 ± 0.06	r = 0.29	0.07
**RBC deformability at 30 Pa (a.u**) $	0.39 ± 0.11	r = 0.27	0.09
**RBC aggregation index (%)**	49.3 ± 10.4	ρ = - 0.17	NS
**RBC disaggregation threshold (s^-1^) §**	278 ± 102	r = - 0.43	< 0.01
**VOC rate**	0.31 ± 0.46	ρ = 0.06	NS
**ACS rate**	0.09 ± 0.16	ρ = - 0.07	NS
**FEV (l)**	1.82 ± 0.61	r = - 0.16	NS
**FVC (l)**	2.01 ± 0.66	r = - 0.16	NS
**FEV/FVC (%)**	91.4 ± 8.6	r = 0.13	NS

Values represent mean ± SD. MAP, mean arterial blood pressure; BMI, Body Mass Index; SpO_2_, arterial hemoglobin oxygen saturation; MCH, mean cell hemoglobin; VOC, vaso-occlusive crises; ACS, acute chest syndrome; FEV, forced expiratory volume in 1 second; FVC, forced vital capacity; § covariates included in the multivariate analysis. $ covariates discarded from the multivariate analysis to avoid co-linearity.

### Univariate analyses

We observed significant correlations between the percentage of predicted distance and the level of fetal hemoglobin, platelets count, hemoglobin level, hematocrit and the RBC disaggregation threshold. The mean arterial blood pressure, leukocytes count, RBC count, lactate dehydrogenase level, RBC deformability at 3 Pa and 30 Pa were not significantly correlated with the percentage of predicted distance, but had a p < 0.20 by linear correlation. Neither the clinical complications studied, i.e., VOC and ACS, nor the pulmonary function tests were correlated with the percentage of the predicted distance.

### Multivariate analyses

To identify the determinants of the 6MWT performance in children with SCA, a multivariate linear regression model was performed. This model included the percentage of predicted distance as dependent variable and fetal hemoglobin, platelets count, hematocrit, white blood cell count, lactate dehydrogenase, mean arterial blood pressure, RBC deformability at 3 Pa and RBC disaggregation threshold as covariates. RBC count, hemoglobin level, and RBC deformability at 30 Pa were not included into the model to avoid co-linearity effects with hematocrit and RBC deformability at 3 Pa.

The overall model was significant (R^²^ = 0.704, df = 8, p < 0.001). The Hct (beta = 1.12; 95% CI 0.33 to 1.90; p < 0.01), fetal hemoglobin level (beta = 1.02; 95% CI 0.52 to 1.53; p < 0.001) and RBC deformability (beta = -0.76; 95% CI -1.46 to -0.06; p < 0.05) were significantly associated with the percentage of predicted distance. Because two SCA patients were asthmatic and under medication, the univariate and multivariate analyses were also done after removing these two children but the results were similar (data not shown).

## Discussion

This is the first study showing an implication of blood rheology on the six-minute walk test performance in children with SCA. We showed that a low level of RBC deformability, a high level of anemia and a low fetal hemoglobin level were independent predictors of a reduced 6MWT performance (i.e., low percentage of predicted 6-min walk distance) in SCA children. 

This work is the first one to show an independent association between RBC deformability and the 6MWT performance in SCA children. This is in agreement with a previous study performed in world-class endurance athletes, which reported an association between RBC deformability and aerobic physical fitness [22]. Ours results support the fact that having higher RBC deformability contributes to a greater 6MWT distance in SCA children. A reduction of RBC deformability increases blood flow resistance into the microcirculation [6] and affects tissue oxygenation [11,15] because RBC with poor deformability cannot easily deform to enter into and flow through the microcirculation and bring oxygen to tissues. Based on these results, it is tempting to hypothesize that medication increasing RBC deformability, such as hydroxyurea, could increase physical capacity in SCA children. The results from Hackney et al [23] support this hypothesis. Moreover, RBC deformability in SCA patients is dependant on the quantity of hemoglobin S into RBCs and higher level of fetal hemoglobin increases the delay time of hemoglobin S polymerization, thus partially inhibiting RBC sickling [24]. Our results also suggest that fetal hemoglobin level plays a key role in exercise capacity in SCA population. 

We recently reported that despite reduced hemoglobin concentration and hematocrit level in SCA patients, the local resting muscle oxygen consumption is comparable between SCA patients and healthy subjects because the local resting microvascular blood flow is increased in the former population [25]. Although the hemodynamic compensation for chronic anemia may be sufficient to normalize muscle oxygen consumption at rest, this is not the case during exercise where the needs for oxygen gradually increase with the intensity. The transition from aerobic to anaerobic metabolism appears earlier during exercise in SCA patients in comparison with healthy subjects [26,27]. Indeed, it is not surprising to observe that hematocrit level was independently associated with the percentage of predicted distance in SCA children, which is in accordance with the previous study of Liem et al [28] The key role of hemoglobin and hematocrit levels in exercise capacity in SCA patients is further supported by studies showing an increase of exercise performance in SCA patients after transfusion [29].

The six-minute walk test is a useful assessor of submaximal exercise ability in patients with SCA [2,3] and has been shown to reflect a certain degree of clinical severity; i.e., mainly in the context of pulmonary hypertension [3]. Because SCA is characterized by abnormalities in lung function, one could expect a contribution of lung dysfunction in the decrease of the 6-min walk distance. However, in agreement with the study of Caboot et al [30], our study does not support this hypothesis. To further extend previous studies on the associations between clinical severity and the exercise capacity in SCA patients, we also tested the associations between the 6-min walk distance and the rate of VOC and ACS but our results failed to demonstrate any association. 

In conclusion, our study demonstrated for the first time that the level of fetal hemoglobin and RBC deformability are predictors of the six-minute-walk test performance in SCA children, and confirmed that the performance during such an exercise is affected by the degree of anemia. The association between the level of fetal hemoglobin and the six-minute-walk test performance suggests that hydroxyurea therapy could be very beneficial for the exercise capacity of SCA patients. This should be considered as another motivation for treating the most severe SCA patients with this therapy. Further studies are needed to test the effects of hydroxyurea on exercise capacity accurately. Moreover, the effects of asthma or exercise-induced asthma were not specifically addressed in this study: future studies should focus on this issue. Finally, a larger study is needed to test the effect of gender on the six-minute-walk test performance.
